# Hypercoiling and Single Umbilical Artery Associated With Fetal Placental Malperfusion and Growth Restriction: A Case Report

**DOI:** 10.1002/ccr3.71862

**Published:** 2026-02-03

**Authors:** Masaya Tanimura, Tomo Yamagata, Moyu Matsui, Yusuke Yamaoka, Kohei Ida, Shohei Nakamura, Miyu Tanaka, Motonori Matsubara

**Affiliations:** ^1^ Department of Obstetrics and Gynecology Toyooka Hospital Toyooka Hyogo Japan; ^2^ Department of Experimental Genome Research Research Institute for Microbial Diseases, Osaka University Suita Osaka Japan

**Keywords:** fetal growth restriction, hypercoiling, single umbilical artery, stillbirth

## Abstract

Hypercoiling of the umbilical cord can lead to fetal growth restriction and stillbirth because the cord is vulnerable to blood flow disturbances caused by external forces. A single umbilical artery (SUA) is a relatively common abnormality; however, isolated SUA is associated with fetal growth restriction and stillbirth. We report a case of fetal growth restriction with both SUA and hypercoiling. A 27‐year‐old primigravida was evaluated at 37 weeks of gestation. Prenatal ultrasonography revealed a small‐for‐gestational‐age fetus with a single umbilical artery and a hypercoiled umbilical cord. Continuous fetal monitoring was initiated upon admission. Cardiotocography demonstrated recurrent prolonged decelerations, necessitating an emergency cesarean section. Placental histopathology revealed fetal vascular malperfusion with hyalinized avascular villi and thrombotic occlusion of one umbilical artery, indicating disturbed fetoplacental circulation likely related to SUA and hypercoiling. The coexistence of hypercoiling and a single umbilical artery was associated with the risk of fetal vascular malperfusion and growth restriction. Careful antenatal surveillance and timely delivery are essential to prevent adverse perinatal outcomes in multiple umbilical cord anomalies.

## Key Clinical Message

Although single umbilical artery and hypercoiling are relatively common cord abnormalities, their coexistence was associated with fetal growth restriction and distress requiring close monitoring and emergency cesarean delivery. Overlapping umbilical cord anomalies can markedly increase perinatal risk, highlighting the need for intensive antenatal surveillance and timely intervention.

## 1 Introduction

Umbilical cord hypercoiling increases the risk of fetal growth restriction (FGR) and intrauterine fetal death (IUFD) due to obstruction of the fetoplacental circulation, as the cord blood flow is susceptible to external compression [1]. In addition, single umbilical artery (SUA) is associated with low birth weight and increased cesarean section and neonatal intensive care unit (NICU) admission rates, even in isolated cases without other anomalies [2]. However, the clinical course and placental histopathological findings of concurrent SUA and hypercoiling have been rarely reported. Here, we report a case of FGR and fetal distress resulting from the coexistence of SUA and hypercoiling, with detailed placental histopathological evaluation.

## 2 Case History/Examination

A 27‐year‐old healthy woman, gravida 1 para 0, presented to our hospital for prenatal care. She underwent routine checkups. The growth of her fetus had previously remained approximately 0 standard deviations (SD) on standard growth charts in Japan, but showed an estimated weight of −0.9 SD at 29 weeks' gestation. At the 37‐week prenatal checkup, her fetus showed an estimated weight of 2013 g (−2.14 SD). SUA and hypercoiling were noted for the first time during her checkup, with an antenatal coiling index of 0.84 coils/cm, consistent with hypercoiling (defined as > 0.3 coils/cm).

**FIGURE 1:**
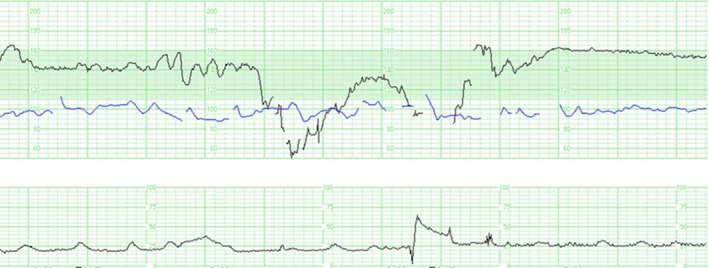
Cardiotocogram at 37 weeks of gestation. The upper black trace shows the fetal heart rate, and the blue trace indicates the maternal heart rate. The lower black trace represents uterine contractions.

The fetal biophysical profile score was 10/10, but cardiotocography (CTG) showed severe prolonged decelerations, which were suggestive of umbilical cord compression (Figure 1). Doppler measurement revealed no absent or reversed end‐diastolic flow of the umbilical artery or brain‐sparing effect. No fetal malformations were observed. The patient was hospitalized for close follow‐up and planned delivery.

## 3 Outcome and Follow‐Up

**FIGURE 2:**
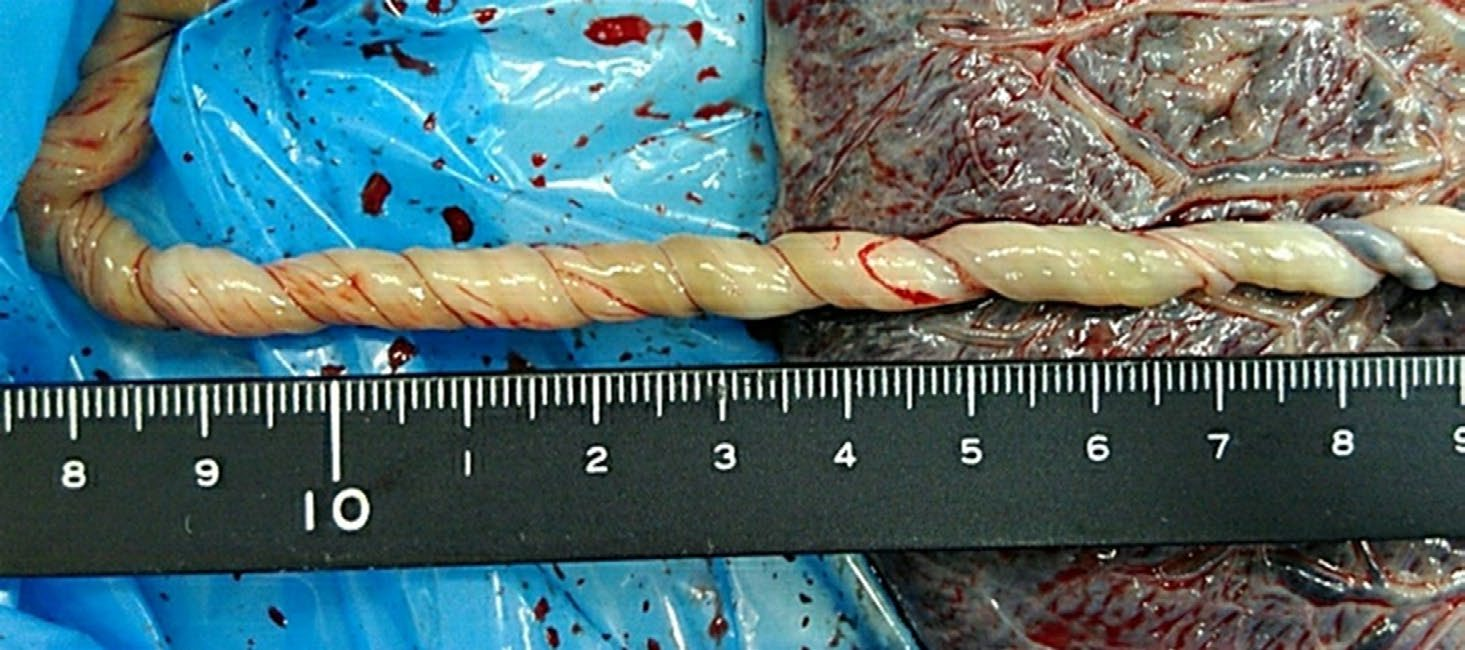
Macroscopic view of the placenta and umbilical cord. The umbilical cord measured 50 cm in length and exhibited 45 coils, fulfilling the criteria for hypercoiling.

During the course of labor induction, prolonged decelerations were observed on cardiotocography. CTG demonstrated recurrent prolonged decelerations, with a nadir of approximately 50 beats per minute and a duration of more than 2 min but < 10 min. Baseline variability was preserved between decelerations. According to the NICHD criteria, these findings were classified as Category II [3, 4]. We diagnosed non‐reassuring fetal status and performed an emergency cesarean section. A male neonate was delivered (1728 g [−2.64 SD]; Apgar scores at 1 and 5 min: 8 and 9, respectively). The umbilical cord length was 50 cm, and the placental weight was 260 g (below the 10th percentile [5]). The number of coils was 45 per 50 cm, which met the diagnostic criteria for hypercoiling (Figure 2). The patient was discharged 6 days after surgery without complications. Blood tests after delivery revealed no signs of infection, coagulation abnormalities, or autoimmune disorders.

Although the neonate was admitted to the NICU because of low birth weight and was examined carefully, his blood tests and head magnetic resonance imaging did not demonstrate any adverse effects during his 30‐day hospitalization.

**FIGURE 3:**
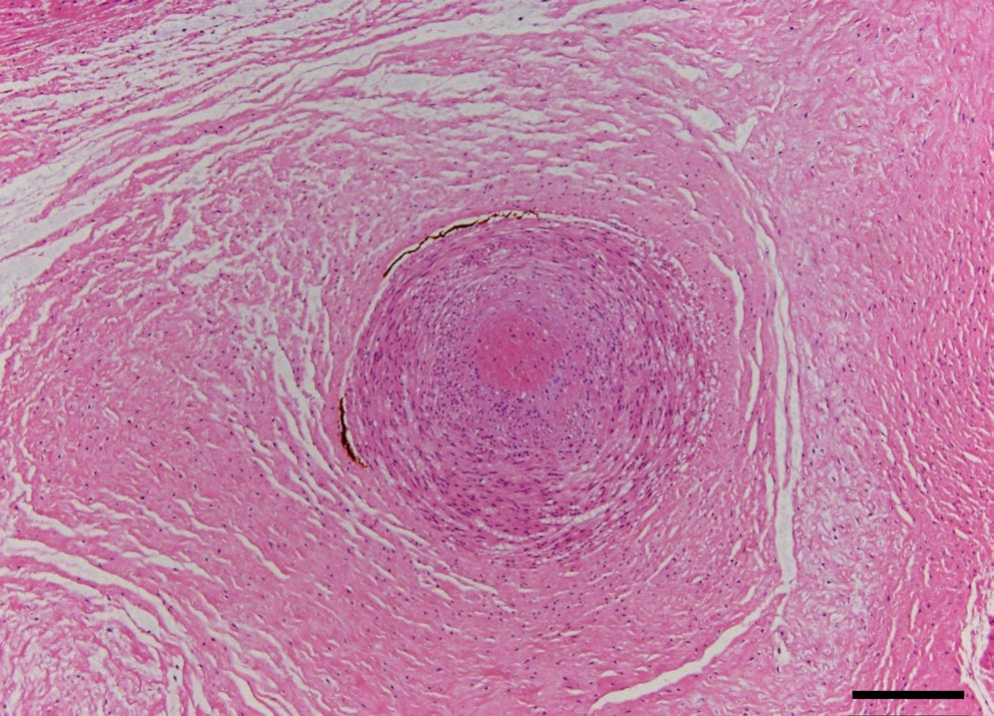
Histologic section showing a single umbilical artery. One of the umbilical arteries is completely occluded by thrombi (hematoxylin and eosin stain). Scale bar = 200 μm.

**FIGURE 4:**
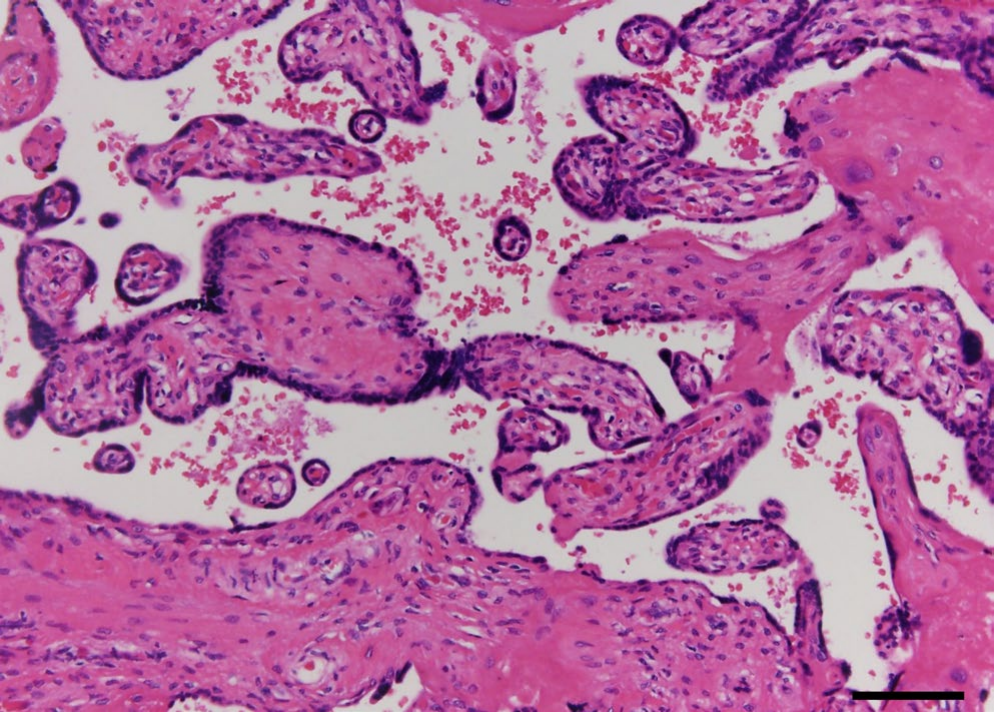
Histologic image of avascular villi. Hyalinized avascular villi showing stromal collagenization and an absence of capillaries. More than 16 avascular villi were observed in this field, consistent with high‐grade fetal vascular malperfusion (hematoxylin and eosin stain). Scale bar = 100 μm.

**FIGURE 5:**
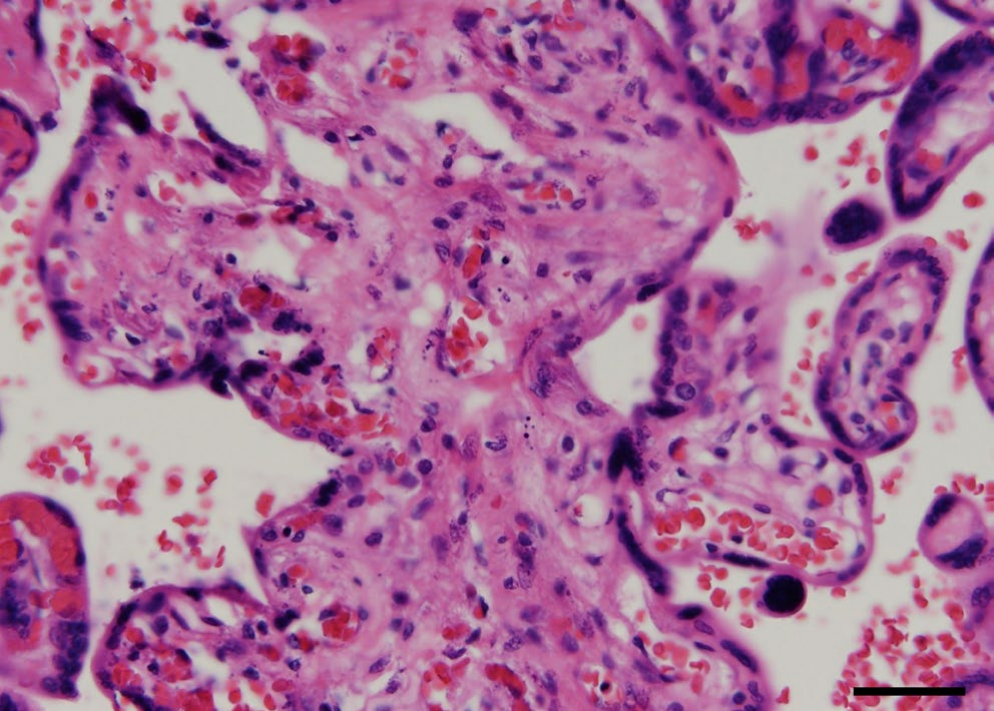
Histologic image of villous stromal vascular karyorrhexis. Distal villi with degenerating stromal tissue containing scattered nuclear debris and red blood cell fragments (hematoxylin and eosin stain). Scale bar = 50 μm.

Histopathological examination confirmed the presence of SUA and fetal vascular malperfusion (FVM). One of the umbilical arteries, which appeared macroscopically occluded, showed transmural necrosis, complete occlusion with thrombi, and atrophy (Figure 3). The placenta exhibited avascular villi (Figure 4), villous stromal vascular karyorrhexis (Figure 5), stem vessel obliteration, and villous mineralization. To assess avascular villi, three histological sections were examined, with an average of > 15 avascular villi per section, consistent with high‐grade FVM (Figure 4). The FVM demonstrated segmental lesions with evidence of widespread involvement of the placental villous tree.

The infant displayed good development and no neurological complications at 1 year of age.

## 4 Discussion

We highlight two key observations in this case. The combination of SUA and hypercoiling may be associated with an increased risk of severe fetal growth restriction and fetal distress. In addition, placental histopathological examination revealed FVM, suggesting chronic circulatory insufficiency.

SUA without other complications was associated with an increased risk of small for gestational age (OR: 2.90, 95% CI: 2.02–4.18) and IUFD (OR: 2.62, 95% CI: 1.43–4.79) [6]. Hypercoiling was also associated with FGR (OR: 6.01, 95% CI: 2.48–15.39) and fetal acidosis (OR: 1.77, 95% CI: 1.16–2.68) [1]. Each of these factors is thought to lead to poor perinatal outcomes via distinct mechanisms: reduced fetoplacental arterial capacity in SUA and intermittent cord compression in hypercoiling. Both factors potentially compromise fetoplacental perfusion. In this case, CTG showed variable decelerations that were suspected to be caused by umbilical cord compression, indicating that a similar circulatory insufficiency may have occurred chronically throughout gestation. This may have caused FGR or nonreassuring fetal status. This pathophysiology was supported by placental histopathological findings of FVM. No other complications were identified, such as major structural anomalies, maternal infections, coagulopathy, or autoimmune diseases that could account for FGR or fetal compromise.

When SUA and hypercoiling occur concurrently, placental histopathology may show more severe FVM. FVM is characterized by lesions such as fetal‐side thrombi/stenosis, stem vessel obliteration, villous stromal–vascular karyorrhexis, and clusters of avascular villi. In the present case, we found clusters of avascular villi, villous stromal–vascular karyorrhexis, stem vessel obliteration, and villous mineralization, indicating chronic obstruction of the umbilical cord vessels. The coexistence of villous stromal–vascular karyorrhexis and clusters of avascular villi, together with stem vessel obliteration and villous mineralization, suggests that fetal vascular obstruction occurred at multiple time points rather than as a single acute event. These findings support an ongoing and recurrent process of fetal vascular malperfusion, with lesions at different stages of evolution, consistent with chronic and intermittent impairment of fetoplacental circulation. As the lesions were multiple and extensive, they were classified as high‐grade FVM [7]. High‐grade FVM is strongly associated with IUFD and neonatal central nervous system abnormalities [8]. Multiple cord abnormalities have also been reported to increase the risk of FVM and fetal hypoxia [9].

Several reports have described FVM associated with isolated SUA, suggesting that SUA itself may increase the risk of FVM and warrants careful monitoring and timely intervention [10]. However, the impact of concurrent SUA and umbilical cord hypercoiling, particularly with respect to the severity of FVM, has not been previously examined. The coexistence of hypercoiling may have predisposed to fetal vascular impairment, leading to more severe placental pathology. The findings in this case suggest that this combination can further compromise fetal circulation and underscore the need for close surveillance, including serial assessment of fetal growth. To our knowledge, this is the first report to describe in detail the clinical course, perinatal outcome, and placental histopathology of a pregnancy complicated by both SUA and hypercoiling, providing important clinical insights into the management of pregnancies with multiple umbilical cord abnormalities.

Although antenatal Doppler indices remained normal in this pregnancy, severe FVM was later confirmed by placental pathology. This highlights that normal Doppler findings do not rule out significant placental vascular pathology. Previous studies in preterm populations have shown that FGR does not necessarily correlate with abnormal umbilical artery Doppler findings, which may partially explain this discrepancy [11]. Nonetheless, there is no clear consensus in the literature, and in our case, it is possible that the single umbilical artery altered Doppler hemodynamics in a way that masked underlying fetoplacental insufficiency.

From a clinical perspective, this case highlights several practical considerations. When multiple umbilical cord abnormalities coexist, the risk of occult fetoplacental insufficiency may be higher than that associated with isolated SUA or isolated hypercoiling. Accordingly, heightened clinical vigilance and a lower threshold for intensified fetal surveillance should be considered. Importantly, this case also underscores that normal antenatal Doppler findings do not reliably exclude significant FVM in the presence of structural umbilical cord abnormalities. In such settings, careful interpretation of cardiotocographic findings and close monitoring during labor or induction may facilitate timely recognition of fetal compromise and appropriate intervention.

SUA may result from either atresia or agenesis. In this case, the SUA was attributed to atresia consistent with complete occlusion and atrophy. To date, it has been unclear whether excessive umbilical cord torsion causes an arterial thrombus to form an occlusion, or whether it develops independently of the thrombus. A review on hypercoiling showed that the condition was strongly associated with SUA (OR 8.25, 95% CI: 2.60–26.12) [12]. However, no detailed reports of the clinical course and pathological findings were found within the scope of our search; therefore, further accumulation of cases is essential to analyze their pathogenesis.

## 5 Conclusion

SUA and hypercoiling can cause FGR or fetal distress. Placental histopathological examination in our case revealed FVM, suggesting a relationship with the clinical findings. When SUA or hypercoiling is detected, other umbilical abnormalities should also be considered. If both are present, careful surveillance (e.g., close antenatal follow‐up and continuous CTG at delivery) is warranted. The coexistence of multiple pathological conditions can lead to poor perinatal outcomes owing to impaired fetal vascular perfusion in the placenta. Further cases are necessary to confirm the association.

## Author Contributions

**Masaya Tanimura:** conceptualization, data curation, formal analysis, writing – original draft, writing – review and editing. **Tomo Yamagata:** writing – review and editing. **Moyu Matsui:** writing – review and editing. **Yusuke Yamaoka:** writing – review and editing. **Kohei Ida:** writing – review and editing. **Shohei Nakamura:** writing – review and editing. **Miyu Tanaka:** writing – review and editing. **Motonori Matsubara:** writing – review and editing.

## Funding

The authors have nothing to report.

## Ethics Statement

The authors have nothing to report.

## Consent

Written informed consent was obtained from the patient to publish this case report and accompanying images, per the journal's patient consent policy.

## Conflicts of Interest

The authors declare no conflicts of interest.

## Data Availability

Data sharing not applicable to this article as no datasets were generated or analyzed during the current study.
